# The mediating effects of mobile phone use on ADHD and educational outcomes: a two-step Mendelian randomisation study

**DOI:** 10.3389/fpsyt.2024.1424082

**Published:** 2024-12-04

**Authors:** Huize Lin, Sitong Yuan, Jinna Yu

**Affiliations:** ^1^ Guang’anmen Hospital, China Academy of Chinese Medical Sciences, Beijing, ;China; ^2^ Monash Neuromodulation Research Unit, Department of Physiotherapy, School of Primary and Allied Health Care, Faculty of Medicine, Nursing and Health Science, Monash University, Melbourne, VIC, ;Australia

**Keywords:** two-step Mendelian randomisation, mediation effect, ADHD, education, mobile screen time

## Abstract

**Objective:**

This study investigates the potential mediating role of mobile phone screen time in the causal relationships between Attention-deficit/hyperactivity disorder (ADHD) and educational attainment. Our analysis explores both the effect of ADHD on educational outcomes and the reverse, i.e., the influence of educational attainment on ADHD risk.

**Method:**

A two-sample Mendelian randomisation (MR) analysis was conducted using genetic instruments from genome-wide association studies (GWAS) of European populations. We employed a two-step MR approach to assess the causal effects between ADHD, mobile phone screen time (both frequency and duration), and educational outcomes, including years of full-time education and college completion. Data from public genome-wide association studies encompassing European populations with sample sizes ranging from 55,374 to 470,941 were utilised.

**Results:**

We found significant causal associations between childhood ADHD and educational attainment, partially mediated by mobile phone screen time. Childhood ADHD was negatively linked to years of full-time education (IVW: OR = 0.93, 95% CI = 0.90-0.97, p = 0.000) and college completion (IVW: OR = 0.97, 95% CI = 0.95-0.98, p = 0.000). Mobile phone use frequency mediated 19.3% of the effect on full-time education (β = -0.158) and 11.9% on college completion (β = -0.084). The duration of phone use mediated 64.8% of the effect on college completion (β = -0.054). When ADHD was the outcome, phone use duration mediated -22.45% of full-time education effects (β = 0.426) and -19.62% of college completion (β = 0.433).

**Conclusion:**

Different MR models reveal the complex mediation role of mobile phone use frequency and duration between ADHD and educational attainment, varying by educational outcome type. Frequency mediates the link between childhood ADHD and full-time education/college completion, while duration significantly impacts ADHD when higher education is the outcome. The notable mediation effect of duration on ADHD underscores the need for further study into screen time’s influence on ADHD and academic achievement across stages.

## 1 Introduction

Attention-deficit/hyperactivity disorder (ADHD) is characterised by a persistent pattern of inattentive, hyperactive and impulsive behaviour; however, its clinical presentation is heterogeneous, with a broad spectrum of severity and symptoms that often overlap with other conditions (1). ADHD stands as the most prevalent psychiatric condition among children and adolescents, with a global prevalence rate of approximately 5% (2). Existing literature reveals that individuals with ADHD tend to exhibit lower academic performance and higher school dropout rates in comparison to their typically developing counterparts (3–5). However, the extent to which mobile phone screen time mediates the relationship between ADHD and education remains unclear.

Concurrently, there has been a substantial surge in the prevalence of ADHD on college campuses over the past few decades. Approximately 2% to 8% of the college student population reports clinically significant ADHD symptomatology, with at least 25% of students with disabilities receiving an ADHD diagnosis (6). These academic difficulties often precipitate the referral of ADHD patients to clinical evaluation.

Rapid technological improvements allow users to condense into the experience a growing variety of fast-paced stimuli accessible at almost any time and place through mobile devices (7). In 1999, the average screen time of 8–18-year-olds was 6.21 hours per day, increasing by 2009 to 7:38 hours (8). The growing amount of children’s leisure time in front of screens has raised concerns regarding associated emotions and behaviour (7, 9, 10). Some research suggests a bidirectional relationship between ADHD symptoms and increased screen time utilisation (9, 11). Children and adolescents aged 6–17, either diagnosed with ADD/ADHD or rated as having attention problems/impulsiveness, were found to have a greater rate of screen time (12). Meanwhile, the high dropout of ADHD is also a worrying question (13). However, whether screen time plays a vital role in the relationship between ADHD and education outcomes still needs to be precise.

Recently, a genome-wide association study (GWAS) on ADHD highlighted evidence of its high heritability with a polygenic architecture (1, 13, 14) and underlined the need for timely detection and improved management of ADHD symptoms. This opens avenues to explore the intricate relationship between genetics and ADHD. Mendelian randomisation (MR) is an approach that employs genetic variants as instrumental variables (IVs) to assess the causal effect of a modifiable exposure on an outcome (14). MR can address certain limitations of randomised controlled trials (RCTs) and observational studies, such as time, cost, medical ethics constraints, and potential issues related to reverse causality and confounding factors (14).

Despite the existence of MR studies examining the relationship between risk factors and ADHD, there has been no prior investigation into the mediating effect of mobile phone screen utilisation on the causal relationship between ADHD and educational outcomes. This study investigates the potential mediating role of mobile phone screen time, mainly its frequency and duration, in the bidirectional causal relationship between ADHD and educational attainment. Special emphasis is placed on the differing effects of frequency and duration of mobile phone use. We used a two-step Mendelian randomisation (MR) approach, leveraging genetic instruments from extensive GWAS datasets to explore whether differences in screen time have a causal impact on ADHD symptoms and educational performance.

## 2 Methods

### 2.1 Mendelian randomisation assumptions

**Figure 1:**
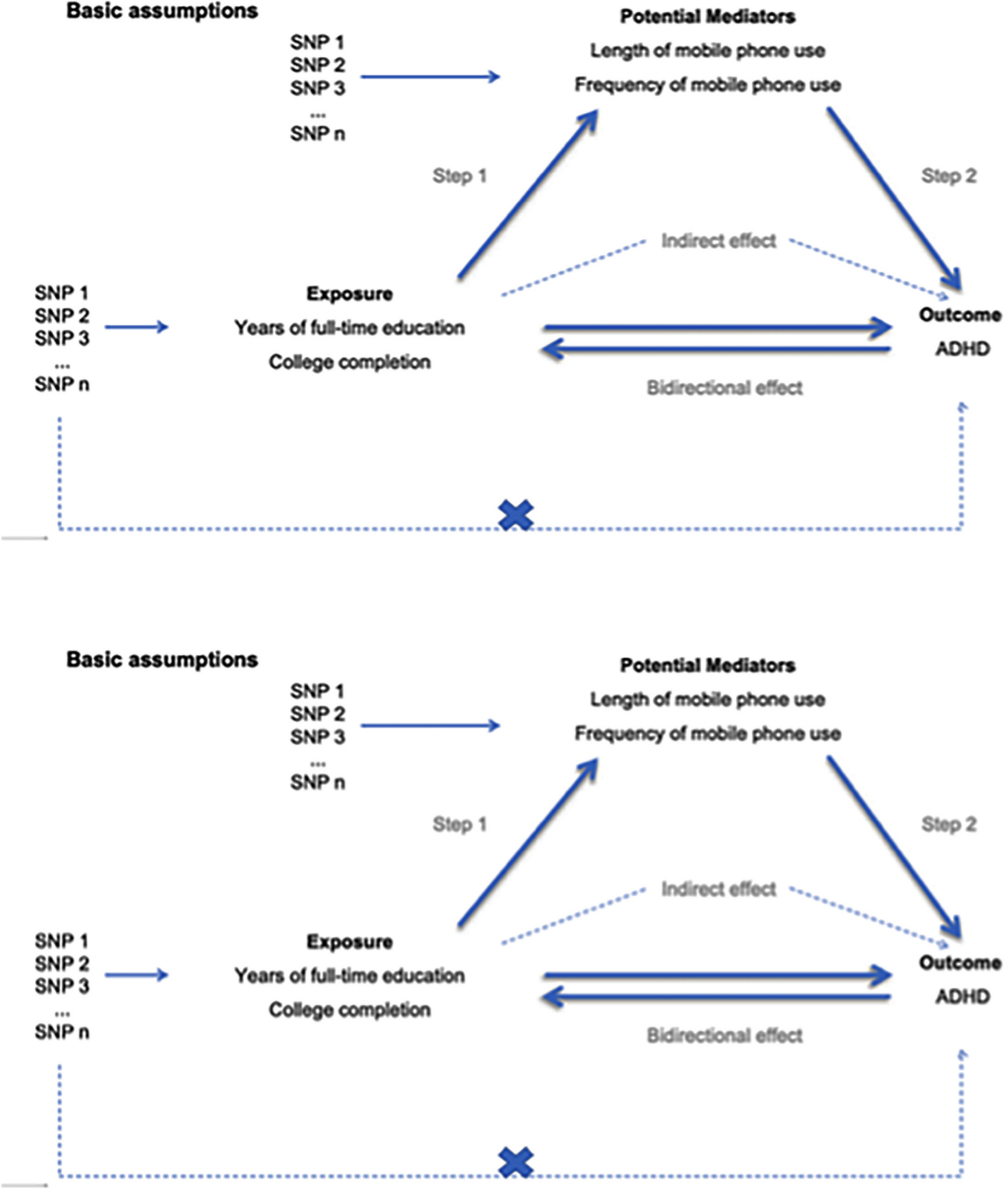
Basic assumptions.

We based our analysis on three key assumptions:

- The relevance assumption: this is crucial in MR, as it stipulates that the genetic variants used as instrumental variables (IVs) must be strongly associated with the exposure (ADHD, mobile phone use frequency, and educational outcomes). Single nucleotide polymorphisms (SNPs) used as IVs will be selected based on genome-wide association studies (GWAS), and the strength of the association will be evaluated using the F-statistic (F > 10) to ensure the avoidance of weak instrument bias.
- The independence assumption: this assumption requires that the genetic instruments not be associated with confounders that influence the exposure and the outcome. We will verify this assumption by conducting bidirectional Mendelian randomisation analyses to test for confounding factors such as socioeconomic status and behavioural variables. The genetic variants used as IVs will be selected to minimise linkage disequilibrium and population stratification, ensuring they are unrelated to potential confounders.
- The exclusion restriction assumption: this assumption stipulates that the genetic instruments influence the outcome solely through exposure (ADHD or educational conditions) and not through any other pathways. We will test for horizontal pleiotropy using MR-Egger regression and Cochran’s Q test. While pleiotropy was not detected in most analyses, caution is warranted due to the potential for residual confounding (**Figure 1**).

### 2.2 Study design

Initially, we conducted bidirectional two-sample MR analyses to explore the relationships between ADHD and educational conditions, including years of full-time education and college completion. Subsequently, a bidirectional two-step MR analysis was performed to investigate potential mediating effects (length and frequency of mobile phone use). In the first step, we conducted univariable bidirectional two-step MR analyses to examine the causal relationships between ADHD, each educational outcome, and each potential mediator. These analyses were conducted independently of the effects of major depression, income, employment status and the Townsend deprivation index (15). In the second step, we performed multivariable MR (MVMR) analyses to investigate the causal relationships between potential mediators, educational outcomes, and ADHD. Our utilisation of bidirectional two-sample MR in this study aims to address mediator bias, particularly in cases where the mediators are interrelated, the outcome affects the mediator, and the instrumental variables influence the mediators through the outcome.

**Figure 2:**
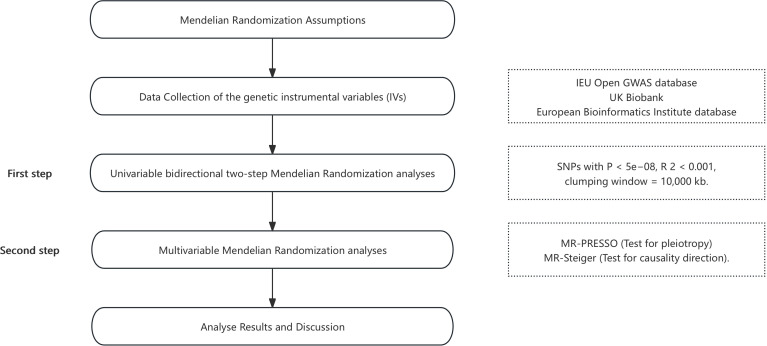
The flowchart of study setup.

A flowchart outlining the study design and analysis strategy was added to clarify the steps involved (**Figure 2**).

#### 2.2.1 Data source and genetic instrumental variables

We performed bidirectional two-sample Mendelian randomisation (MR) analyses using summary-level data from the IEU Open GWAS database (https://gwas.mrcieu.ac.uk/), UK Biobank (https://www.ukbiobank.ac.uk), and the European Bioinformatics Institute database (https://www.ebi.ac.uk). ADHD diagnosis in this study was based on the iPSYCH sample, focusing on children diagnosed using the ICD-10 criteria (F90.0), as recorded in the Danish National Psychiatric Central Research Register (16). However, specific information regarding the age at which screen time exposure occurred was unavailable. These data were specific to individuals of European ancestry and were employed for our MR analysis. All original studies obtained ethical approval (**Table 1**). We used datasets containing the most significant number of single nucleotide polymorphisms (SNPs). All relevant SNPs in each dataset satisfied the significance threshold (P < 5e−08). To ensure that the IVs were not in linkage disequilibrium (LD), we carried out clumping (R ^2^ < 0.001, clumping window = 10,000 kb). The instrument strength for SNPs in MR was assessed by approximated F statistics; IVs with F statistics much more significant than 10 for the instrument-exposure association were considered free from weak instrumental variable bias.

**Table 1:** Summary of SNP information.

Trait	Database ID	Year	Sample size	nSNP
Exposure				
ADHD	ieu-a-1183	2017	55,374	8,047,420
Potential Mediator (Screen time)				
Length of mobile phone use	ukb-b-4094	2018	456,972	9,851,867
Frequency of mobile phone use	ukb-b-17999	2018	386,626	9,851,867
Outcome (Education outcomes)				
Years of full-time education	ukb-a-505	2017	226,899	10,894,596
College completion	ebi-a-GCST90029012	2018	470,941	11,972,619

##### 2.2.1.1 Exposure

We selected the dataset with the most significant number of SNPs for ADHD in individuals of European ancestry from the IEU Open GWAS database, encompassing 55,374 participants.

##### 2.2.1.2 Potential mediator

The screen time data came from self-reported UK Biobank Assessment Center (ACE) questionnaires. In the UKB database, we utilised two variables related to mobile phone screen time exposure. The genetic IVs for the length of mobile phone use included data from 456,972 individuals. As for the frequency of mobile phone use, we employed the measure of weekly phone usage in the last three months, involving 386,626 individuals.

##### 2.2.1.3 Outcome

We considered two distinct variables related to educational attainment as education outcomes. The first variable was the years after full-time education (questionnaire-based, available only for individuals who did not pursue degree-level education) (17). The genetic IVs for age-completed full-time education were sourced from the UKB database, encompassing 226,899 participants. The second educational attainment variable was college completion, and the corresponding genetic IVs were obtained from EBI databases, including data from 470,941 participants.

### 2.3 Statistical analysis

We used several MR techniques to evaluate the causal effects of ADHD, mobile phone screen time (frequency and duration), and educational attainment. To assess the strength of the association between genetic instruments and phenotypes, we reported the proportion of variation in exposure and all mediators explained by their genetic variant instruments. Additionally, we calculated the F-statistic for the regression of exposure, outcome and all mediators on their genetic instruments. We conducted all statistical analyses using R Studio (Copyright (C) 2022 by Posit Software, PBC, version 2023.09.0 + 463) software.

The primary outcome was based on IVW, assuming that all genetic instrumental variables are valid or that horizontal pleiotropic effects of instruments are balanced (18). MR-Egger assumed that the impact of the instrument on the exposure is not correlated with a direct effect of the instrument on the outcome (19). Weighted Median assumed that no more than 50% of the weight of the estimate is from invalid genetic instrumental variables (18). We also conducted a leave-one-out analysis to assess whether MR estimates were influenced or biased by individual SNPs.

#### 2.3.1 Heterogeneity and pleiotropy testing

Cochran’s Q test was used to assess heterogeneity, with significance set at p < 0.05, indicating the presence of notable heterogeneity in the genetic instruments. The MR-Egger regression intercept test was used to identify horizontal pleiotropy. A significant MR-Egger intercept (p < 0.05) would indicate directional pleiotropy, meaning that genetic variants affect the outcome through pathways other than the exposure of interest. The leave-one-out method was also applied to determine whether a single genetic variant drove the results. The IVW method was used for the primary analyses as it assumes that all genetic instruments are valid unless horizontal pleiotropy is detected.

#### 2.3.2 Mediation analysis

The proportion of the total effect mediated by mobile phone screen time was estimated using the product of coefficients method. Specifically, we multiplied the effect estimate of ADHD on mobile phone screen time by the effect estimate of mobile phone screen time on educational outcomes. The significance of the indirect effect (i.e., mediation effect) was assessed using the delta method to calculate standard errors and 95% confidence intervals (CIs) for the mediated effect.

For the mediation analysis, we reported the proportion of mediation, p-values, effect sizes, and 95% confidence intervals for all significant indirect effects.

#### 2.3.3 Sensitivity analysis

To ensure the robustness of our findings, we conducted multiple sensitivity analyses. These included MR-Egger, which allows for some invalid instruments, and Weighted Median, which provides consistent estimates if more than 50% of the weight comes from valid instruments. In addition, we employed the MR-PRESSO (Pleiotropy RESidual Sum and Outlier) method to detect and correct for horizontal pleiotropy by identifying outlier SNPs. Only SNPs passing the horizontal pleiotropy tests were retained for final analyses.

## 3 Results

**Figure 3:**
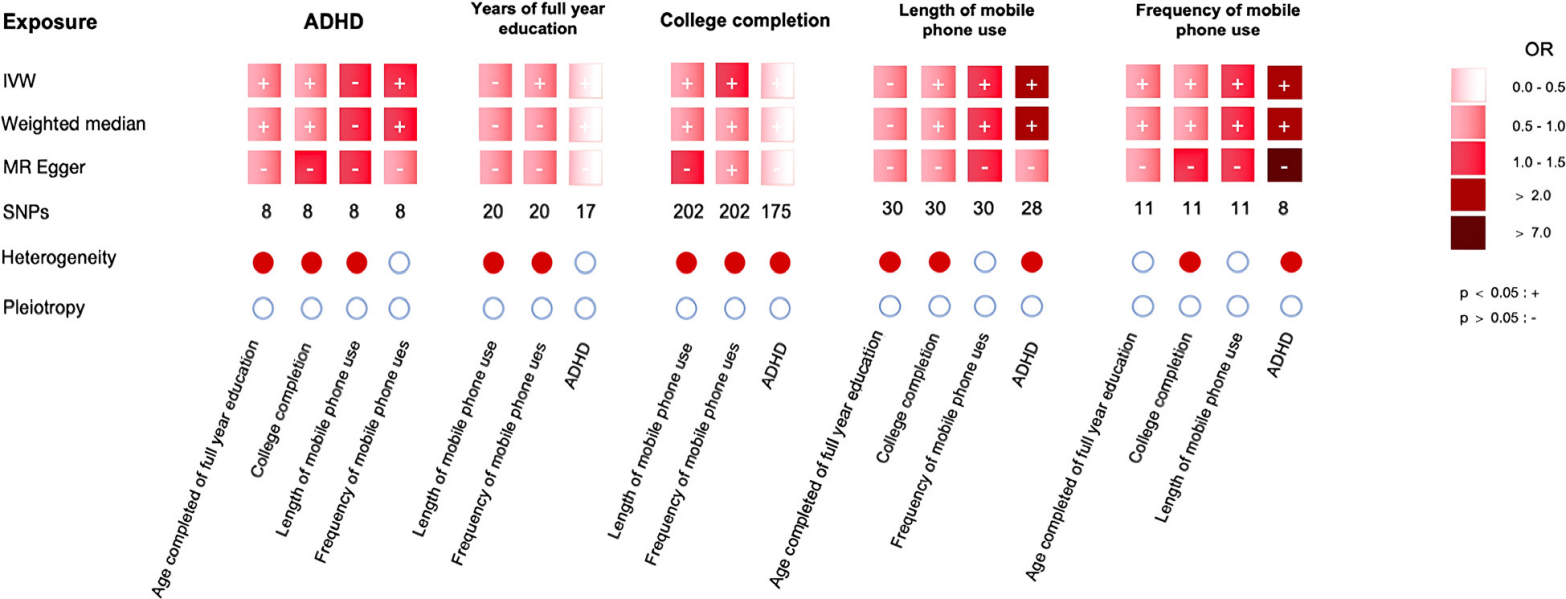
The main results.

In our study, we performed bi-directional MR analyses to evaluate the exposure (ADHD/education outcomes), the potential mediation (mobile phone screen time), and the outcome (education outcomes/ADHD) (**Figure 3**). SNPs associated with the exposure and potential mediators were considered instrumental variables (IVs) for MR analysis if they met the criteria of p < 5 × 10^−8, LD (R ^2^) > 0.01, kb < 10,000, and F > 10.

### 3.1 Bidirectional univariable MR analyses between ADHD and educational outcomes

We conducted bidirectional two-sample MR analyses on independent SNPs to estimate the causal relationships between childhood ADHD and years of full-time education and college completion. In this study, the diagnosis of childhood ADHD was based on the ICD-10 criteria, with data sourced from the iPSYCH sample (16).

When considering ADHD as the exposure and educational outcomes as the outcomes, an increased risk of childhood ADHD showed a negative causal association with years of full-time education (IVW: OR = 0.93, 95% CI = 0.90 to 0.97, p = 0.000) and college completion (IVW: OR = 0.97, 95% CI = 0.95 to 0.98, p = 0.000) (**Figure 3**). This indicates that a higher risk of ADHD is negatively associated with both full-time education duration and college completion. Cochran’s Q test revealed heterogeneity among the SNPs related to years of full-time education (Q = 20.3, p < 0.001) and college completion (Q = 18.7, p = 0.002). We also used MR-PRESSO to identify and exclude outlier SNPs, and the re-analysis yielded results consistent with the original analysis. MR-Egger regression did not show significant horizontal pleiotropy (intercept p > 0.05), indicating that the genetic instruments mainly affected the outcome through exposure. The results of the weighted median method (OR = 0.93, 95% CI = 0.90-0.97, p = 0.000) were consistent with those of MR-Egger and IVW. However, as our ADHD data is based on a pediatric cohort (16), these findings underscore the impact of ADHD on educational attainment in early developmental stages.

In the analysis where years of full-time education were considered the exposure and childhood ADHD as the outcome, the results showed a negative causal association between increased years of full-time education and childhood ADHD risk (IVW: OR = 0.14, 95% CI = 0.08 to 0.23, p = 0.000) (**Figure 3**). MR-Egger regression did not show significant horizontal pleiotropy (intercept p > 0.05), indicating that the genetic instruments primarily influenced the outcome through the exposure. The results of the weighted median method were consistent with those of MR-Egger and IVW (OR = 0.14, 95% CI = 0.08-0.23, p = 0.000) (**Figure 3**).

When analysing college completion as the exposure and childhood ADHD as the outcome, the results demonstrated a significant negative causal association between higher rates of college completion and childhood ADHD risk (IVW: OR = 0.12, 95% CI = 0.07 to 0.18, p = 0.000) (see **Figure 3**). However, there was heterogeneity among the SNPs related to college completion and ADHD (MR-Egger p = 0.000, IVW p = 0.000), although MR-Egger regression did not detect horizontal pleiotropy (Egger intercept = -0.008, p = 0.245) (**Figure 3**). The MR-PRESSO also did not reveal significant pleiotropy (p = 0.12). After removing the outlier SNPs, the re-analysis yielded results consistent with the original analysis (**Figure 3**).

The leave-one-out method demonstrated that removing any SNP did not fundamentally affect the relationship between education outcomes/ADHD risk and ADHD risk/education outcomes.

### 3.2 Mediation analysis

We performed a two-step MR analysis to assess the potential mediating role between exposure (ADHD/education outcomes) and outcome (education outcomes/ADHD).

#### 3.2.1 First step: two-sample MR

We examined the associations between exposures, potential mediators, and outcomes (**Figure 3**).

In our analysis, childhood ADHD was causally associated with the frequency of mobile phone use (IVW: OR = 1.06, 95% CI = 1.03 to 1.10, p = 0.000). While no significant causal relationship was found between ADHD and the length of mobile phone use in most analyses (IVW: OR = 1.01 95% CI = 0.97 to 1.05 p = 0.735), certain models indicated a potential causal association, which warrants further investigation. This suggests that frequent use, rather than the total duration, plays a more prominent role in the ADHD-education relationship. There was no evidence of heterogeneity (MR Egger p = 0.225, IVW p = 0.183) and horizontal multiplicity (Egger intercept = 0.007, p = 0.28). These findings suggest that frequent mobile phone use in children diagnosed with ADHD may have a more significant impact on their education than duration of use.

When years of full-time education were the exposure, we found that the associations were statistically non-significant with years of full-time education and length (IVW: OR = 1.01, 95% CI = 0.97 to 1.05, p = 0.735) and frequency of mobile phone use (only IVW method p < 0.05: OR = 1.06, 95% CI = 1.03 to 1.10, p = 0.000), indicating no significant association between genetically determined years of full-time education and the mobile phone screen time use condition. For the analysis of educational attainment as the exposure, we found that educational attainment was causally associated with both the length (IVW: OR = 0.78, 95% CI = 0.70 to 0.86, p = 0.000) and frequency (IVW: OR = 0.61, 95% CI = 0.55 to 0.67, p = 0.000) of mobile phone use. All results displayed heterogeneity (MR Egger p < 0.05, IVW p < 0.05), and no horizontal multiplicity was observed (all p > 0.05).

When analysing the length of mobile phone use as the exposure, we found that it has causal associations with ADHD (IVW: OR = 2.01, 95% CI = 1.31 to 3.08, p = 0.001) and college completion (IVW: OR = 0.92, 95% CI = 0.88 to 0.96, p = 0.000) but not with years of full-time education (IVW: OR = 0.93, 95% CI = 0.86 to 1.00, p = 0.065). All results displayed heterogeneity (MR Egger p < 0.05, IVW p < 0.05), and no horizontal multiplicity was observed (all p > 0.05).

For the analysis of the frequency of mobile phone use as the exposure, we found that the frequency of mobile phone use was causally associated with ADHD (IVW: OR = 2.54, 95% CI = 1.02 to 6.35, p = 0.045), years of full-time education (IVW: OR = 0.80, 95% CI = 0.74 to 0.88, p = 0.000), and college completion (IVW: OR = 0.87, 95% CI = 0.80 to 0.94, p = 0.001). Heterogeneity was absent in the frequency of mobile phone use and years of full-time education analysis results (MR Egger p = 0.225, IVW p = 0.254). In contrast, heterogeneity was present in other analysis results (all p > 0.05).

We also examined the relationship between the length and frequency of mobile phone use to estimate bias, and the results indicated a bidirectional relationship, the results in **Figure 3**.

#### 3.2.2 Second step MVMR

In the second step, we conducted multivariable MR (MVMR) analyses to evaluate the causal relationships between mobile phone use (frequency and duration), childhood ADHD, and educational outcomes. We used the same instrumental variables (IVs) as in the univariable MR analyses. Specifically, SNPs that met the genome-wide significance threshold (p < 5e-08) and were not in linkage disequilibrium (R² < 0.001, clumping window = 10000 kb) were selected as IVs for both univariable and MVMR analyses.

For the analysis with years of full-time education as the outcome, we identified that 44 SNPs have causal associations between age of completed full-time education and childhood ADHD (IVW OR = 0.93, 95% CI: 0.89 to 0.97, p = 0.000) as well as frequency of mobile phone use (IVW OR = 0.78, 95% CI: 0.71 to 0.86, p = 0.000), but not length of mobile phone use (IVW OR = 0.94, 95% CI: 0.87 to 1.01, p = 0.091). The results suggest that frequent phone use plays a more prominent role in affecting educational outcomes in children diagnosed with ADHD compared to the duration of use.

Similarly, we use college completion as an outcome to establish causal links with 44 SNPs. Additionally, we found it has causal relationships with ADHD (IVW OR = 0.96, 95% CI: 0.94 to 0.98, p = 0.001), frequency (IVW OR = 0.85, 95% CI: 0.79 to 0.92, p = 0.000), and length of mobile phone use (IVW OR = 0.92, 95% CI: 0.83 to 0.98, p = 0.000).

In scenarios where the years of full-time education, length, and frequency of mobile phone use served as exposure, 47 SNPs may unveil causal connections. The results demonstrated that years of full-time education (IVW OR = 0.15, 95% CI: 0.08 to 0.27, p = 0.000) and the length of mobile phone use (IVW OR = 1.92, 95% CI: 1.15 to 3.21, p = 0.013) exhibited causal associations with ADHD. However, we observed no significant associations with the frequency of mobile phone use (IVW OR = 2.62, 95% CI: 0.93 to 7.40, p = 0.068).

In analysing college completion, length, and frequency of mobile phone use as exposure, we employed 187 SNPs to explore causal relationships. The findings indicated that college completion (IVW OR = 0.11, 95% CI: 0.07 to 0.18, p = 0.000) and the length of mobile phone use (IVW OR = 1.94, 95% CI: 1.13 to 3.34, p = 0.017) were causally linked to ADHD, whereas no significant association was found with the frequency of mobile phone use (IVW OR = 3.06, 95% CI: 0.74 to 12.66, p = 0.123)—the results of heterogeneity tests and pleiotropy tests in **Tables 2**, **3**.

**Table 2:** Educational condition used as MVMR outcome-heterogeneity tests and pleiotropy tests.

		Years of full-time education		College completion	
MV exposures	Methods	SNPs	P value	SNPs	P value
ADHD	MR Egger	7	0.007	7	0
	IVW	7	0.005	7	0
	Egger intercept	/	0.409	/	0.145
Length of mobile phone use	MR Egger	29	0	29	0
	IVW	29	0	29	0
	Egger intercept	/	0.556	/	0.728
Frequency of mobile phone use	MR Egger	8	0.289	8	0
	IVW	8	0.389	8	0
	Egger intercept	/	0.868	/	0.628

**Table 3:** ADHD as MVMR outcome-heterogeneity tests and pleiotropy tests.

		ADHD					
MV exposures	Methods	SNPs	P value	MV exposures	Methods	SNPs	P value
Years of full-time education	MR Egger	15	0.071	College completion	MR Egger	169	0
	IVW	15	0.069		IVM	169	0
	Egger intercept	/	0.378		Egger intercept	/	0.223
Length of mobile phone use	MR Egger	25	0	Length of mobile phone use	MR Egger	14	0.010
	IVW	25	0		IVM	14	0.014
	Egger intercept	/	0.247		Egger intercept	/	0.676
Frequency of mobile phone use	MR Egger	7	0	Frequency of mobile phone use	MR Egger	4	0.001
	IVW	7	0.001		IVM	4	0.003
	Egger intercept	/	0.834		Egger intercept	/	0.731

#### 3.2.3 Mediating effect

When full-time education was the outcome, MVMR analysis showed a significant association between mobile phone use frequency and ADHD. In contrast, the duration of mobile phone use did not show a significant association. When higher education was the outcome, both mobile phone use frequency and duration showed significant associations. However, when ADHD was the outcome, only mobile phone use duration was significantly associated with both full-time and higher education exposure. Therefore, we conducted mediation effect analyses on the parts with significant associations in the MVMR analysis to explore the impact of screen time on ADHD and educational outcomes.

We used the product of coefficients method to estimate the indirect effects. When educational outcomes were considered as the result, the mediating role of mobile phone use frequency significantly influenced the relationship between childhood ADHD and years of full-time education (β = -0.158, mediation proportion = 19.3%), as well as the relationship between childhood ADHD and higher education completion (β = -0.084, mediation proportion = 11.9%) (**Table 4**). Additionally, the mediating role of mobile phone use duration significantly impacted the relationship between ADHD and higher education completion (β = -0.054, mediation proportion = 64.8%) (**Table 4**). When ADHD was the outcome, the mediation effect of mobile phone use duration was -22,45% (β=0.426) when full-time education was the exposure and -19.62% (β=0.433) when completion of college was the exposure (**Table 5**).

**Table 4:** Mediation effect of frequency of mobile phone use on educational outcome.

Outcome	Years of full-time education					College completion				
Mediator	Total effect (β)	Direct effect 1	Direct effect 2	Indirect effect (β)	Mediation proportion	Total effect (β)	Direct effect 1	Direct effect 2	Indirect effect (β)	Mediation proportion
Frequency of mobile phone use	-0.035	0.059	-0.217	-0.158	19.3%	-0.035	0.059	-0.143	-0.084	11.9%
Length of mobile phone use	/	/	/	/	/	2.7264	0.96	0.92	1.7664	64.8%

**Table 5:** Mediation effect of Length of mobile phone use on ADHD.

	Outcome	ADHD				
Exposure	Mediator	Total effect (β)	Direct effect 1	Direct effect 2	Indirect effect (β)	Mediation proportion
Years of full-time education	Length of mobile phone use	-1.897	0.653	0.653	0.426	-22.45%
College completion		-2.207	0.663	0.653	0.433	-19.62

## 4 Discussion

ADHD patients often exhibit academic functioning impairment, putting them at a higher risk for academic underachievement and dropout (20–22). Systematic reviews and meta-analyses have identified small yet significant correlations between screen time and ADHD (23, 24). However, until recently, literature regarding children’s and adolescents’ digital media use has been chiefly related to TV and computer use (7). In contrast, more children and adolescents have shifted toward using mobile devices (7). To follow this trend, our study applied two-sample bidirectional Mendelian randomisation (MR) and multivariable MR (MVMR) methods to explore the complex causal relationships between mobile screen use duration and frequency, educational outcomes, and ADHD. We aim to assess the potential mediating role of mobile screen use frequency and duration between ADHD and educational outcomes. Based on extensive genetic data from the IEU Open GWAS, UK Biobank, and the European Bioinformatics Institute, we employed multiple statistical methods, including IVW, MR Egger, and the weighted median approach, along with MR Egger and MR-PRESSO sensitivity analyses to ensure the robustness of the results and to minimise pleiotropy’s impact on causal inferences.

In the first step of the study, we analysed the bidirectional causal relationship between ADHD and educational outcomes. Our results are consistent with previous studies (25), indicating that ADHD negatively impacts educational outcomes, while longer participation in education may help reduce the risk of ADHD symptoms. It is important to note that the data in this study were derived from a pediatric population, so these findings should be interpreted cautiously when applying them to adult ADHD cases.

In the second step of the MVMR analysis, different results were obtained between the univariable MR and MVMR models during the mediation effect analysis despite conducting heterogeneity and pleiotropy tests. However, in contrast to previous studies on the effects of screen time on ADHD, this study also emphasises the effects of prolonged screen time on ADHD (26). In this study, the differences are reflected in two main aspects. First, there is a discrepancy in the causal association between mobile phone use frequency and ADHD. In the univariable MR analysis, mobile phone use frequency was significantly associated with ADHD, but this association became non-significant in the MVMR analysis. This difference might stem from the methodological distinctions between the approaches (27). Univariable MR considers only a single exposure variable, while MVMR includes multiple exposure variables, allowing for better control of potential confounding effects among these variables (19). In the univariable MR analysis, mobile phone use frequency may be associated with the duration of screen use, thus exhibiting a significant causal relationship. However, in MVMR, when mobile phone use duration is simultaneously considered, the effect of frequency might be masked, leading to a non-significant association.

Additionally, the wider confidence interval in the MVMR analysis (95% CI = 0.93 to 7.40) suggests that the estimate for mobile phone use frequency might be unstable, possibly due to interaction effects between exposure variables or insufficient statistical power. Second, the causal effect of mobile phone use duration on ADHD varies in strength. The univariable MR analysis showed no significant causal association between mobile phone use duration and ADHD. However, in the MVMR analysis, mobile phone use duration was significantly associated with ADHD. Similar to the mobile phone use frequency situation, this difference may result from the different exposures controlled in the respective models. In the univariable MR, the impact of mobile phone use duration might have been masked by frequency. Alternatively, the increase or decrease in mobile phone use duration may not proportionally affect the outcome but instead show varying effect patterns depending on the duration (28). The MVMR analysis can control for these potential factors, providing a more nuanced understanding of the complex relationship between mobile phone use duration and ADHD under multiple exposures (29, 30). Future studies should investigate the distinct impacts of frequency and duration on ADHD further and consider the complex interactions between these factors.

The fact that different models yield different results does not necessarily indicate a contradiction. Instead, it may reflect the complex mechanisms underlying the data (27). One possible explanation is that the effect of mobile phone use duration on ADHD or educational outcomes may follow a complex, non-linear pattern (31). It is also possible that other risk factors may influence the outcomes, even though some potential confounders (including major depression, income, employment status and the Townsend deprivation index (15)) were controlled for in the study. Some evidence suggests that ADHD risk is associated with both genetic and non-genetic environmental risk factors, and the digital media landscape could be one of these environmental factors (32, 33). Individuals with ADHD tend to be more sensitive to immediate reward feedback and have lower self-control, which could increase their inclination toward longer and more frequent screen time use in a digital media context (28, 34).

The effect of screen time on ADHD is cumulative (12, 28), with studies indicating a dose-response relationship between screen time and ADHD symptom severity (35). Excessive use of digital devices and media may affect brain function and cognitive development (23), which is linked to the severity of ADHD symptoms (36). Recent MRI studies found a connection between long-term screen use and ADHD risk (11, 37), mediated by changes in the microstructure of white matter in the brain (38). Screen time has a direct adverse impact on early childhood brain development. Therefore, it is essential to consider the critical role of screen time more effectively (12). Current research on the effects of screen time on the cognitive and psychological health of adolescents and adults is still in its early stages (39), and the neurodevelopmental mechanisms and consequences remain incompletely understood (36, 39). In particular, there is a lack of research on screen time as a mediating factor in adult ADHD and its neurodevelopmental mechanisms (32, 40).

It is important to note that, although our study suggests a potential link between mobile phone use and the development of ADHD, the limited data reflecting only the past three months of screen time restrict our ability to draw definitive conclusions about its long-term impact on brain development. Furthermore, due to the lack of age-specific screen time data, we cannot determine at which developmental stage mobile phone use may have the greatest impact.

Future research should focus on exploring the cumulative effects of screen time over extended periods and across different developmental stages to gain a more comprehensive understanding of its impact on ADHD and brain development. Collecting long-term and age-specific screen time exposure data to investigate its influence in more detail is equally important.

## 5 Limitation and future studies

This study has several limitations worth considering.

Firstly, the data for this analysis were derived from a cohort of children with ADHD. In contrast, the screen time exposure data from the UKB database predominantly includes middle-aged and elderly participants (41). Although the genetic instruments used in the Mendelian Randomisation approach are stable across the lifespan (42), the age disparity between the genetic data and the screen time exposure may limit the generalizability of the findings to younger cohorts.

Secondly, the screen time exposure data only reflects usage patterns over the past three months. This short observation period restricts our ability to assess the effects of screen time on brain development at different critical stages. Some studies suggest that frequent mobile phone use could have long-term implications across different age groups, warranting further study (11, 37).

Thirdly, the length and frequency of mobile phone use relied on self-reports and parent reports, potentially leading to underestimation and subjectivity. The models did not account for confounding factors such as parental sensitivity and parent-child interactions, which will be addressed in future studies.

Finally, although we applied several methods to detect and correct for pleiotropy, completely ruling out pleiotropic effects remains a significant challenge in Mendelian randomisation studies. Additionally, residual confounding and the inability to fully capture genetic and environmental factors’ complexity may limit our causal inferences’ robustness.

## 6 Conclusion

Different MR models reveal complexity in these causal links. This study shows that mobile phone use frequency and duration serve as partial mediators in the relationship between ADHD and educational attainment, with nuanced effects depending on the type of educational outcome. Specifically, mobile phone use frequency mediates the link between childhood ADHD and full-time education and college completion. In contrast, phone use duration significantly impacts ADHD when higher education is the outcome. The mediation effect of phone use duration is notably substantial for ADHD outcomes, emphasising the need for further investigation into how screen time influences ADHD and academic achievement across developmental stages.

## Data Availability

The original contributions presented in the study are included in the article/supplementary material. Further inquiries can be directed to the corresponding author.
